# Clinical efficacy of single intraoperative 500 mg methylprednisolone management therapy for thoracic myelopathy caused by ossification of the ligamentum flavum

**DOI:** 10.1186/s12891-020-03216-2

**Published:** 2020-03-19

**Authors:** Xiaoyang Huo, Jiaming Zhou, Shiwei Liu, Xing Guo, Yuan Xue

**Affiliations:** 1 Department of Orthopedic Surgery, Tianjin Medical University General Hospital, Tianjin, China; 2 Tianjin Key Laboratory of Spine and Spinal Cord, Tianjin Medical University, Tianjin, China

**Keywords:** Methylprednisolone, Ossification of the ligamentum flavum, Thoracic spine surgery, Clinical outcome, Surgical site infection

## Abstract

**Background:**

The objective of our study was to compare clinical outcome and postoperative complications between patients with thoracic myelopathy caused by ossification of the ligamentum flavum (OLF) treated with and without intraoperative methylprednisolone (MP).

**Methods:**

This retrospective study enrolled 101 patients who underwent posterior approach surgery for OLF and were followed up at least 1 year. Patients were divided into two groups according to MP use in the operation: MP group (*n* = 47) and non-MP group (*n* = 54). Clinical outcomes and complications were evaluated before and after operation and at the last follow-up.

**Results:**

Significant differences were found in modified Japanese Orthopedics Association (mJOA) scores and proportion of Frankel grade (A-C) between the two groups immediately after surgery and at 2-week follow-up. No significant differences were found between the two groups in mJOA score before operation and at the final follow-up. Moreover, no significant differences were observed in recovery rate according to mJOA score at any time points, and there was no significant difference in the proportion of Frankel grade (A-C) between the two groups at final follow-up. There were 13 documented infections: 10 in the MP group and 3 in the non-MP group (*P* = 0.034).

**Conclusion:**

Management therapy with intraoperative 500 mg MP showed better recovery of nerve function within 2 weeks in patients with thoracic myelopathy caused by OLF compared with those did not receive MP. However, long-term follow-up results showed that there was no significant difference in neurological recovery between patients with intraoperative MP or not. Moreover, intraoperative MP increased the rate of wound infection.

## Background

Ossification of the ligamentum flavum (OLF) has been widely regarded by clinicians as a primary reason for thoracic myelopathy in Asia [1]. The major pathological mechanism of OLF is local compression of the ligament [1]. For patients with thoracic myelopathy unresponsive to conservative treatment, surgery is a routine treatment. To achieve favorable clinical results, the area around the spinal cord must be fully decompressed [2, 3]. Posterior laminectomy decompression to remove the hypertrophic and ossified ligamentum flavum is the most common surgical procedure in thoracic spine surgery for the treatment of OLF [4]. However, the thoracic spinal canal is very narrow compared to the cervical spinal canal and lumbar spinal canal, and blood flow in the thoracic spinal cord is weak [5]. In addition, the ossified ligament often sticks to the dura mater, making the operation very difficult [5, 6]. The postoperative recovery of functional outcome is also poor [1].

Methylprednisolone (MP) is a steroid that has been widely used in various clinical diseases owing to its potent anti-inflammatory effect. When administered within the first 8 h after traumatic spinal cord injury, MP has been reported to improve neurological outcomes and short-term motor scores [7]. Nevertheless, evidence suggests that steroids compromise the peripheral immune system regardless of injury or injury conditions [8–10]. Pia et al. verified that in mouse models, MP protects neurons from inflammation by not damaging a portion of circulating immune cells, thereby reducing perioperative neurological complications following decompression of cervical myelopathy. Hence, they recommend that MP should be considered as a perioperative management therapy to alleviate neurological complications associated with decompression surgery [11]. However, the effect of intraoperative steroid application in spine surgery has been doubted by some clinicians [12, 13].

The use of intraoperative steroids remains debatable in spine surgery. Furthermore, its effect in thoracic myelopathy caused by OLF is unknown. Hence, the aim of this study was to elucidate the clinical outcome of intraoperative MP on the efficacy of posterior approach surgery for treating thoracic myelopathy caused by OLF, as observed over a minimum 1 year of follow-up.

## Methods

### Population

Between December 2009 and December 2017, under approval of our institutional review board, a retrospective study was conducted involving patients with thoracic OLF who underwent posterior decompression. All patients were selected from a common referral pool, and the surgeries were performed by the same team. Patients with thoracic OLF who underwent posterior decompression and fusion were included in the study. The exclusion criteria included patients who underwent revision surgery, staged surgery, or surgery due to infection or malignancy. Patients who were unavailable for follow-up, pregnant women, dialysis patients, and chemotherapy patients were also excluded. Computed tomography (CT) was used to divide OLF into unilateral, bilateral, or bridged type. Sagittal magnetic resonance imaging (MRI) classified OLF into round or beak shape [14]. T2-weighted MRI was used before surgery to determine the target area for decompression. Postoperative modified Japanese Orthopedics Association (mJOA) score and proportion of Frankel grade (A-C) were assessed.

### Surgical protocol

Surgeries were performed by the same team consisting of three spine surgeons in our hospital. Support staff, operating rooms, surgery equipment, and post-anesthesia care unit were uniform. The patients received perioperative antibiotic therapy based on the standard process at 1 h before the incision, and a weight-based dose of cefazolin was provided. For patients allergic to cephalosporin, clindamycin was used. Between December 2009 to March 2015, patients with surgery were administrated MP intraoperatively; after March 2015, patients treated by surgery were not given MP. Hence, patients were divided into two groups after surgery: the MP (*n* = 47 [46.5%]) and non-MP (*n* = 54 [53.5%]) groups. For patients in the MP group, a single dose of 500 mg MP was given via peripheral vein before the laminectomy, and the process was completed within 1 h. All patients were performed an open surgery with or without the use of an operative microscope.

### Data collection and clinical assessment

Data on patients’ demographics, comorbidities, clinical outcomes, and postoperative hospitalization complications were collected. Demographic items included age, sex, body mass index, smoking history, and alcohol consumption history. Comorbidities included hypertension and diabetes. Operative characteristics included blood loss, operative time, intraoperative transfusion, and number of operated thoracic levels. Postoperative complications included surgical site infection (SSI), pneumonia, deep vein thrombosis, neurologic worsening, and cerebrospinal fluid leak. mJOA scores and Frankel grading scores were evaluated at clinical follow-up.

### Statistical analysis

Data analysis was performed using Statistical Package for the Social Sciences (SPSS version 20.0, Chicago, IL). Data are reported as mean with standard deviation. Unpaired two-tailed Student’s *t*-test or Wilcoxon test were used to compare continuous data, and Fisher’s exact test was used to classify data. All tests were two-sided and significant if the *P* < 0.05.

## Results

**Fig. 1:**
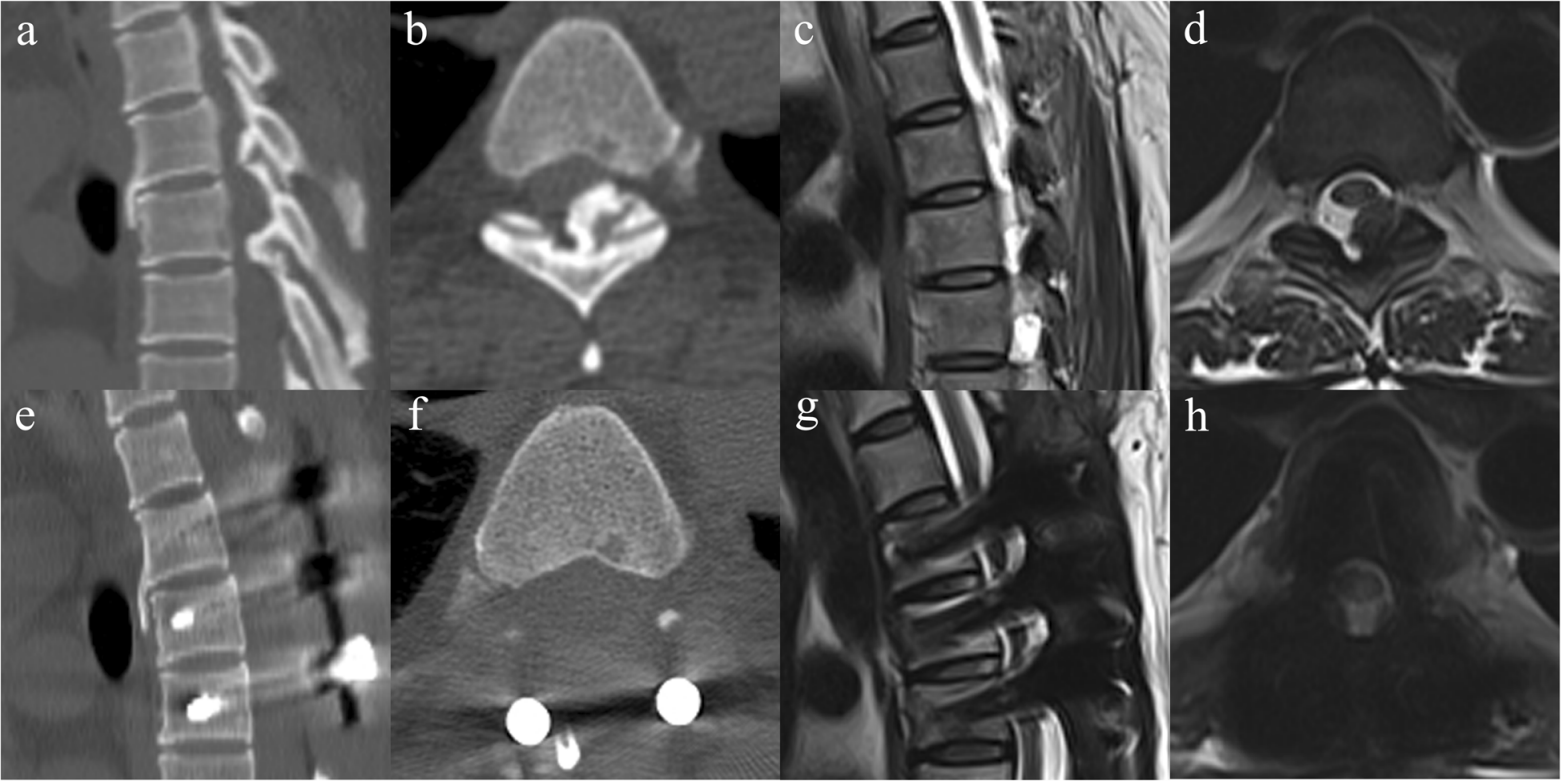
A patient with OLF received intraoperative MP. Preoperative CT and MRI show OLF at T3-T5 (**a-d**). The larger ossified ligament flavum (T4-T5) is unilateral (**b**, axial CT) and round type (**c**, sagittal MRI), with no T2 high intensity at corresponding spinal cord on MRI (**d**, axial MRI). Postoperative CT and MRI demonstrate good decompression (**e-h**). One week after surgery, wound infection was found. Bacteria culture result was methicillin-sensitive *Staphylococcus aureus*. Treatment methods were intravenous vancomycin and local wound care. The patient then made a full recovery without further infection symptoms

Patient characteristics are summarized in Table 1. A total of 101 patients were recruited in our study, including 47 patients who received MP (MP group) and 54 patients who did not receive MP (non-MP group). No significant differences in demographics and comorbid characteristics were observed between both groups. Also, there is no obvious difference in OLF types and intensity change on MRI between two groups (Table 2). In addition, both groups corresponded well in terms of surgical complexity (Table 3). A patient with OLF in MP group was shown in Fig. 1.

**Table 1:** Characteristics of patients undergoing thoracic spine surgery

Items	MP (*n* = 47)	Non-MP(*n* = 54)	*P*-Value
Age	47.47 ± 13.36	51.07 ± 16.19	0.348
Male	26 (55.3%)	31 (57.4%)	0.843
BMI	23.04 ± 5.85	24.02 ± 5.69	0.837
Hypertension	36 (76.6%)	39 (72.2%)	0.655
Diabetes	12 (25.5%)	11 (20.1%)	0.636
Smoking history	24 (64.9%)	26 (48.1%)	0.843
Alcohol history	31 (66.0%)	31 (57.4%)	0.418

Values are reported as number (percent) or mean ± standard deviation. *BMI* body mass index

**Table 2:** OLF type and Intensity change in MRI of spinal cord

Items	MP (*n* = 47)	Non-MP(*n* = 54)	*P*-Value
Type (axial CT)			
Unilateral	24(51%)	23(43%)	0.329
Bilateral	18(38%)	28(52%)	
Bridged	5(11%)	3(6%)	
Type (sagittal MRI)			
Beak	9(19%)	7(13%)	0.426
Round	38(81%)	47(87%)	
T2 high Intensity	34(72%)	32(59%)	0.210

Values are reported as number (percent). *OLF* ossification of the ligamentum flavum, *CT* computed tomography, *MRI* magnetic resonance imaging

**Table 3:** Surgical characteristics

Items	MP (*n* = 47)	Non-MP (*n* = 54)	*P*-Value
Operative time (min)	186.38 ± 77.44	192.22 ± 62.95	0.148
Blood loss (ml)	469.79 ± 95.61	453.28 ± 106.8	0.687
Intra-op transfusion	8 (17.0%)	11 (20.4%)	0.800
No. of operated thoracic levels	3.89 ± 0.84	4.20 ± 0.87	0.481

Values are reported as number (percent) or mean ± standard deviation. *Intra-op* intraoperative, *no. of* number of

The clinical outcomes of the patients are listed in Table 4. For mJOA scores, significant differences between the two groups were observed immediately after surgery and at the 2-week follow-up. Moreover, the proportions of Frankel grade (A-C) scores were significantly higher in the MP group than in the non-MP group immediately after surgery and at the 2-week follow-up. No significant differences in mJOA and Frankel scores were observed before surgery and at the last follow-up. The duration of follow-up months for the two groups were 18.00 ± 3.69 months and 17.72 ± 3.51 months, respectively (*P* = 0.758).

**Table 4:** Clinical outcome

Items	MP (*n* = 47)	Non-MP (*n* = 54)	*P*-Value
mJOA			
Preop	4.36 ± 1.71	4.02 ± 1.84	0.626
Postop Immediately	6.43 ± 2.50	4.96 ± 2.00	**0.036**
2-weeks FU	7.49 ± 1.93	6.43 ± 2.49	**0.039**
Final follow-up	9.02 ± 1.70	9.06 ± 1.71	0.677
mJOA recovery rate (%)			
Final FU	70.07 ± 23.39	71.15 ± 25.40	0.949
Frankel grade (A-C)			
Preop	40 (85.1%)	44 (81.5%)	0.791
Postop Immediately	20 (42.3%)	35 (64.8%)	**0.029**
2-weeks FU	17 (36.2%)	32 (59.3%)	**0.031**
Final FU	13 (27.7%)	13 (24.1%)	0.820
Duration of FU	18.00 ± 3.69	17.72 ± 3.51	0.758

Values are reported as number (percent) or mean ± standard deviation. *mJOA* modified Japanese orthopedics association. *Preop preoperative* Postop postoperative, *FU* follow-up

Significant differences (*P* < 0.05) are marked in bold

Postoperative complications are listed in Table 5. There were 13 documented infections: 10 in the MP group and 3 in the non-MP group (*P* = 0.034). There were no significant differences between the two groups in the incidence of other complications, such as deep vein thrombosis (*P* = 1.000), pneumonia (*P* = 0.413), neurologic worsening (*P* = 0.672), and cerebrospinal fluid leak (*P* = 0.644). Durotomies in both groups were mainly repaired by microsurgery. All infection cases were cured by antibiotics combined with wound drainage.

**Table 5:** Postoperative complications

Items	MP (*n* = 47)	Non-MP (*n* = 54)	*P*-Value
SSI	10 (21.2%)	3 (5.6%)	**0.034**
DVT	3 (6.4%)	3 (5.6%)	1.000
Pneumonia	4 (8.5%)	2 (3.7%)	0.413
Neurologic worsening	2 (4.3%)	3 (3.7%)	0.672
CSF leak	10(21.2%)	14(25.9)	0.644

Values are reported as number (percent). *SSI* surgical site infection, *DVT* deep vein thrombosis. *CSF* cerebrospinal fluid

Significant differences (*P* < 0.05) are marked in bold

## Discussion

OLF is a primary reason for thoracic myelopathy, and it can result in paralysis of the lower extremities in severe cases. It usually causes blunt spinal compression, and conservative treatment is usually ineffective. Surgery is the only effective method to treat OLF [15]. However, surgical intervention of the thoracic spine has high incidence of complications [16, 17]. SSI after posterior thoracic spine surgery is the most common complication and the reason for revision surgery. The incidence of postoperative SSI ranges from less than 3% in discectomy and laminectomy to approximately 12% in instrument fusion surgery [18]. The effect of SSI on morbidity and clinical outcome cannot be ignored [19, 20]. Our study found that intraoperative administration of 500 mg MP accelerated the 2-week neurological recovery of patients with thoracic myelopathy due to OLF. However, an increase in infection rate was also observed in those who received intraoperative 500 mg MP.

Steroids can alleviate inflammatory responses by inhibiting chemotactic accumulation of inflammatory cells, adhesion of leukocytes, and release of histamine and kinins. Steroids have been shown to reduce phospholipase A2 activity, inhibit nociceptive C fiber conduction, stabilize cell membranes, and inhibit prostaglandin synthesis [21]. MP is the least irritant and the most effective steroids, with the longest time [22]. There is an evidence that in spinal surgery, steroids reduce neuropathic pain by preventing spontaneous nerve discharge from injured nerves [23]. In addition, preoperative steroids can repair systemic inflammatory responses and inhibit iatrogenic defects [24]. Furthermore, it has been reported that steroids protect neurons from inflammation without impairing the composition of circulating immune cells, thereby reducing perioperative neurological complications following cervical decompression surgery [11]. The mechanism of action of steroids is not fully understood. Thus, future studies are required to advance our understanding of this mechanism [25].

Although the effect of management with intraoperative steroids on postoperative complications and prognosis of spinal surgery is still unclear, some spine studies found no correlation between steroid use and better postoperative outcomes. For example, a prospective study by Bednar et al. [13] indicated that patients treated with and without intraoperative steroid showed no significant difference in wound healing or infection rate. In fact, some studies have shown negative postoperative prognosis associated with intraoperative steroid treatment. Christian et al. [12] found that intraoperative steroid management had no effect on the postoperative outcome of cervical spine surgery, and that it increased the rate of wound infection. Similarly, our study confirmed that intraoperative steroid administration had no effect on the long-term outcome of thoracic spine surgery, and that SSI rate was higher in patients who received intraoperative steroid than in those who did not. However, unlike previous studies, the present study also found that steroids promoted the recovery of neurological function within 2 weeks.

On the contrary, perioperative and intraoperative applications of steroids were found to be effective in several studies. For example, a prospective study reported that patients treated with perioperative steroids showed significant improvement in short-term and long-term functional outcomes [26]. In addition, Song et al. [27] indicated that short-term use of systemic MP after anterior cervical discectomy and fusion was effective in reducing dysphagia and reducing prevertebral soft tissue swelling. Moreover, short-term application of MP was not associated with postoperative infection. Furthermore, Anders et al. [26] found that steroid treatment reduced pain and improved functional outcome and prolonged hospital stay after microscopic disc surgery. An analogous study showed that patients receiving steroids for lumbar decompression or cervical radiculopathy had shorter hospital stay and less postoperative pain [28]. Similarly, our study showed that the recovery of neurological function in patients treated with intraoperative MP was accelerated within 2 weeks.

In our study, better neurological outcomes were found in MP group at short-term follow-up, but no significant differences were observed between the two groups at long-term follow-up, indicating that MP may only contribute to short-term recovery of spinal cord function. This phenomenon could be partially explained by the short half-life of MP [11, 29]. In a meta-analysis of MP in spinal cord injury, the authors enrolled two RCTs and concluded that MP was associated with a significant motor score improvement at short-term follow-up but not at long-term follow-up [7]. However, for observational studies subgroup analysis, no positive associations were found between MP and motor score improvement at short-term or long-term follow-up [7]. In addition, for postoperative neurologic worsening in our study, no statistical difference was found between the two groups, making this short-term effect of neurological recovery of MP more limited.

Nevertheless, this study had several limitations. First, owing to the retrospective nature of this study, all items were retrospectively collected and analyzed; thus, the analysis was weak. Second, the small sample size could have generated selection bias. Despite these limitations, however, our study indicated that intraoperative MP administration accelerated the recovery of spinal cord function within 2 weeks after posterior approach surgery for OLF, but also increased postoperative infection rate.

## Conclusion

In posterior approach surgery for OLF, intraoperative MP administration showed better recovery of neurological function within 2 weeks after surgery, but had no significant difference on the long-term outcome compared with those no MP received. Moreover, intraoperative MP treatment obviously increased SSI rate.

## Data Availability

The datasets analyzed during the current study are available as a supporting file or from the corresponding author on reasonable request.

## Abbreviations

BMI: Body mass index
CSF: Cerebrospinal fluid
CT: Computed tomography
DVT: Deep vein thrombosis
FU: Follow-up
intra-op: Intraoperative
mJOA: Modified Japanese orthopedics association
MP: Methylprednisolone
MRI: Magnetic resonance imaging
no. of: Number of
OLF: Ossification of the ligamentum flavum
preop: Preoperative; postop: postoperative
SSI: Surgical site infection
